# Comparative analysis of the effect of Ca and Mg ions on antibacterial activity of lactic acid bacteria isolates and their associations depending on cultivation conditions

**DOI:** 10.1186/s13568-019-0758-9

**Published:** 2019-02-28

**Authors:** Lusine Matevosyan, Inga Bazukyan, Armen Trchounian

**Affiliations:** Department of Biochemistry, Microbiology and Biotechnology, Faculty of Biology, Yerevan State University, 1 Alex Manoogian Str., 0025 Yerevan, Armenia

**Keywords:** Lactic acid bacteria, LAB associations, Antibacterial activity, Ca and Mg ions

## Abstract

The effects of divalent cations of Ca and Mg on antibacterial activity of lactic acid bacteria (LAB) isolates, as well as their different associations were studied. Most LAB strains and associations revealed significant inhibitory effects in MRS against Gram-positive and Gram-negative test-organisms at different Ca^2+^ and Mg^2+^ concentrations (determined specifically for each LAB strain and each association). Some LAB strains and communities inhibited the growth of pathogenic test-organisms depending on both ions concentrations and cultivation conditions. Interestingly, the presence of Mg ions in medium significantly decreased the antimicrobial activity of LAB communities against pathogenic test-organisms; on the other hand, the combined mixture of ions essentially increased the inhibitory effect in case of time-spaced cultivation. In contrast, the inhibitory effects of many associations were significantly increased at the presence of Mg^2+^ and especially ions combination in case of simultaneous cultivation. The addition of ions combination didn’t affect antibacterial activity of LAB isolates. The results allow us to conclude that Ca and Mg ions had inducible effects on antibacterial activity in case of simultaneous cultivation. This probably can be prospective for creation of new antimicrobial preparations and their possible application.

## Introduction

The reduction of pathogens growth in food and feed production and storage is very important, and the creation of new effective strategies for this purpose becomes more and more prospective. It has been known that lactic acid bacteria (LAB) produce several antimicrobial substances, including organic acids, other organic compounds, carbon dioxide, diacetyl, hydrogen peroxide and bacteriocins (Nes et al. 2011). It is also significant to define the role of metals in production of antimicrobial components and differentiate the metals that are essential for cells and included in composition of important enzymes (proteases, DNA polymerases, dipeptidases, tripeptidases, etc.). Such metals are calcium (Ca^2+^) and magnesium (Mg^2+^).

Investigations of the role of metals in microbial growth delayed because procedures for purification and detection were not sensitive enough to measure the small amounts of ions generally required by bacteria. Besides these technical troubles, circumstantial studies on the mineral demands for the growth of organisms are complicated because of the following (Boyaval 1989): metals replace each other; some metals adsorb others; some metals interact differently in the presence of others; many organic substances can combine with metals and render them unavailable for growth; colloidal suspensions of metals can precipitate because of shifts in pH before or during growth.

The great multiplicity of ecological-geographic conditions of Armenia with its precisely designated vertical zoning contributes the development of unique associations of LAB in traditional dairy products. During centuries Armenians have prepared traditional protein-enriched dairy products such as yoghurt, sour cream and different types of cheeses, having substantial physiological, antagonistic, antioxidant and antiallergenic activity (Bazukyan et al. 2010; Movsesyan et al. 2010). Antibacterial activity is connected to a synthesis of special substances-bacteriocins (Nes et al. 2012). Bacteriocin producing LAB can be applied as a starter cultures in food fermentation or added to a fresh food as bio-preservatives.

The investigation of different metals’ role in bacteria growth and antagonistic activity becomes more and more popular and required in recent years. It is interesting to study the change of inhibitory activity of bacteria by addition to their growth medium ions of different metals and compare the results. It has been showed that the antibacterial activity of LAB pure cultures could be differing from the same activity of the LAB associations, which is connected to the types of cultivation.

Consequently, the aim of this work was to compare the effects of Ca^2+^ and Mg^2+^, as well as their combined mixture on antibacterial activity of LAB isolates and their associations depending on cultivation conditions.

## Materials and methods

### Objects of investigation

The objects of investigation were different LAB strains isolated from dairy products and gastrointestinal tract of honeybees: *Lactobacillus rhamnosus* R-2002 (the Accession Number is KY054594 and submitted in GenBank) deposited at Microbial Depository Center (MDC) (WDM803) (‘Armbiotechnology’ Scientific and Production Center, National Academy of Sciences of Armenia, Yerevan, Armenia) under the number MDC9661, *Lactobacillus delbrueckii* subsp. *bulgaricus* (RIN-2003-Ls)*, L. delbrueckii* subsp. *lactis* INRA-2010-4.2 and *L. delbrueckii* subsp. *bulgaricus* INRA-2010-5.2 under the code numbers MDC9632 and MDC9633, respectively (Keryan et al. 2017), *Streptococcus thermophilus* VKPM B-3809*, Enterococcus durans* (provided by Institut Nationale de la Recherche Agronomique, Nantes, France, INRA), *Lactobacillus delbrueckii* subsp. *bulgaricus* B7 (the accession number is MK494928 and submitted in GenBank).

### Creation of LAB associations

For creation of associations LAB strains were cultivated in modified MRS broth at 37 °C during 24 h. Before starting an experiment, isolates were mixed at equal proportions (1:1) according to mathematical planning of experiments (Table 1) (Goers et al. 2014). In each combination, the amount of LAB was corrected till 10^8^ CFU/mL.

**Table 1:**
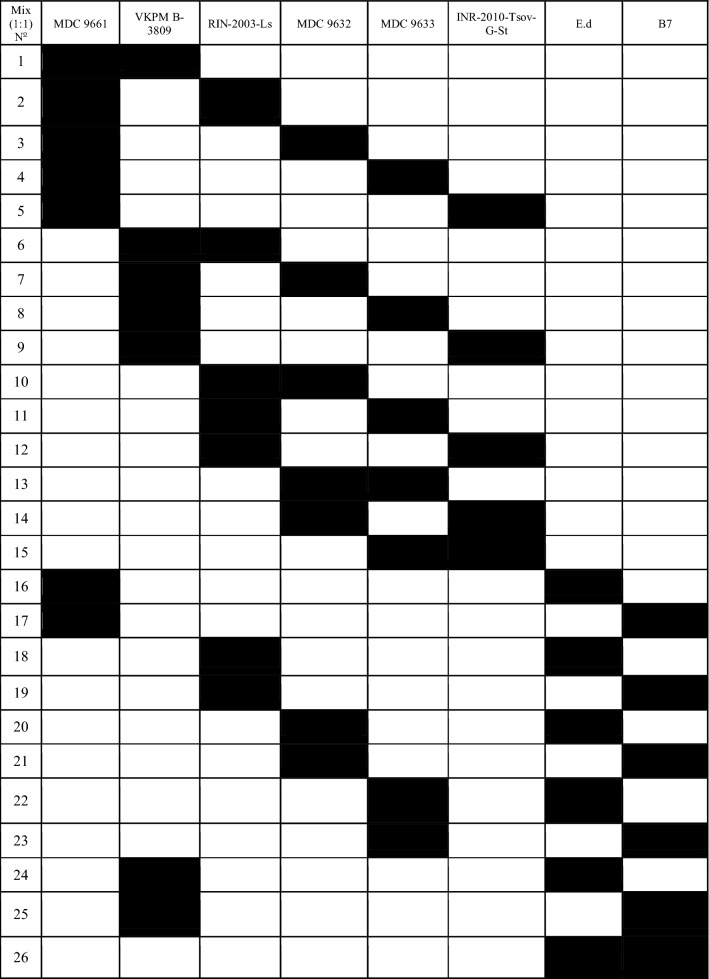
LAB associations (mixes)

### Investigation of effect of Ca^2+^ and Mg^2+^ on biological properties of lactic acid bacteria and their mixes

To determine the stimulating effects of Ca^2+^ and Mg^2+^ on LAB antibacterial activity the primary screening was carried out. As a source of ions CaCl_2_ and MgCl_2_ salts were used. For study of metal ions effects on LAB biological properties MRS medium was prepared containing 5, 8, 10 and 12 mM Ca^2+^ and Mg^2+^. The optimal concentration of metal ions for each LAB strain and association was determined. Simultaneous effect of two ions in optimal concentrations on LAB antibacterial activity was studied, too.

### Determination of antibacterial activity

The antibacterial activity of 7 LAB strains was carried out previously (Keryan et al. 2017; Movsesyan et al. 2010). The antibacterial activity of 7 LAB isolates and 21 associations in MRS at different concentrations of Ca and Mg ions was determined by agar well diffusion method (Ndagano et al. 2011). Various groups of microorganisms were used as test-organisms: *Escherichia coli* VKPM-M17 (Russian National Collection of Industrial Microorganisms, Institute of Genetics and Selection of Industrial Microorganisms, Moscow, Russia), *Staphylococcus aureus* MDC5233, *Salmonella typhimurium* MDC1759, *Pseudomonas aeruginosa* WT272786 (isolated from clinical material and provided by “Prom-Test” LLC, Yerevan, Armenia), *Bacillus mesentericus* WT, *B. subtilis* WT-A1 (isolated from a soil sample) and *Micrococcus luteus* WT (isolated from an air sample). Determination of the preferable concentration of metals for inhibitory effects of LAB isolates was investigated at first. Then the antagonistic activity of associations at the presence of metal ions was carried out, too.

The effect of metal ions on antagonistic activity of LAB communities was carried out by 2 ways of cultivation (time-spaced and simultaneous cultivation of LAB strains at 37 °C). In case of time-spaced cultivation, the LAB isolates were incubated in MRS with the best concentration of Ca^2+^ and Mg^2+^ or their combined mixture and then the LAB mixtures were combined. Each well was filled with 50 μL of each overnight LAB cultural liquid (2 different LAB strains). Then Petri dishes were placed at room temperature for 30 min diffusion of antibacterial substances. After 24 h of incubation at the optimum temperature required for the test-culture growth, diameters of growth inhibition zones were measured. A clear zone of inhibition of at least 2 mm in diameter was recorded, as positive. In case of simultaneous cultivation, 2 separate LAB strains were added at equal proportions into the same growth medium (MRS-broth) with the best average concentration of Ca^2+^ and Mg^2+^ or their combined mixture and cultivated together at 37 °C during 24 h. 0.1 mL of overnight mixed culture was added to wells and tested for antibacterial activity.

### Data processing

All data were averages of three independent experiments. The standard errors were determined using Software Excel 2013.

## Results

### The effect of Ca and Mg ions on antibacterial activity of LAB isolates

**Fig. 1:**
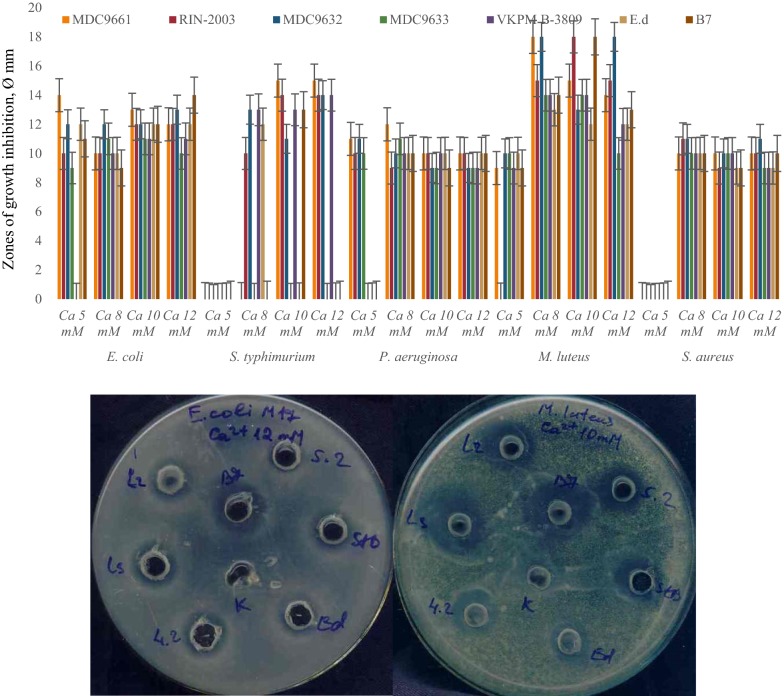
The effect of different concentrations of Ca^2+^ on antibacterial activity of LAB isolates

**Fig. 2:**
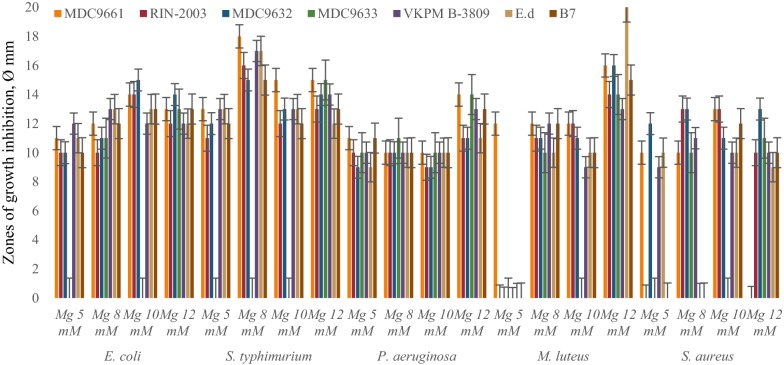
The effect of different concentrations of Mg^2+^ on antibacterial activity of LAB isolates

**Fig. 3:**
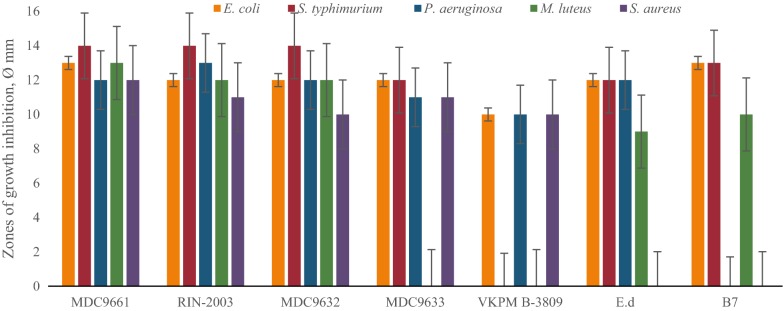
The effect of combined mixture of Ca^2+^ and Mg^2+^ on antibacterial activity of LAB isolates

Primary screening of LAB revealed that the most active strains are *L. rhamnosus* R-2002 (KY054594), *L. delbrueckii* subsp. *bulgaricus* (RIN-2003-Ls), *L. delbrueckii* subsp. *lactis* (INRA-2010-4.2), *L. crispatus* (INRA-2010-5.2), *S. thermophilus* (VKPM B-3809), *E. durans*, *L. delbrueckii* subsp. *bulgaricus* (B7). That is why it was interesting to study the effects of divalent cations of Ca and Mg, as well as their combined mixture on antimicrobial activity of LAB isolates. The results of these investigations indicated the following findings. Most LAB strains revealed significant inhibitory effect against *E. coli* and *M. luteus* in case of addition to their growth medium 10 mM CaCl_2_, while INRA-2010-4.2 and INRA-2010-5.2 showed their best activity at 12 mM and 8 mM of CaCl_2_, respectively (Fig. 1). The antimicrobial activity of investigated LAB strains was significantly increased in case of addition Mg ions to MRS (Fig. 2). Particularly, R-2002, RIN-2003-Ls and *E. durans* showed their strongest inhibitory effect against *E. coli*, *S. typhimurium*, *M. luteus* and *S. aureus* at 10 mM of MgCl_2_. For antimicrobial activity of INRA-2010-4.2, INRA-2010-5.2 and B7 the preferable concentration of MgCl_2_ was 12 mM and for VKPM B-3809 8 mM, respectively. Interestingly, the addition of 12 mM MgCl_2_ induced the inhibitory effect of INRA-2010-5.2 and B7 even against the pathogenic *P. aeruginosa*. In all cases the studied LAB strains indicated the static effect against *B. mesentericus*. No any activity was observed against metal-resistant *B. subtilis* WT-A1 strain. The addition of combined mixture of Ca^2+^ and Mg^2+^ to the LAB growth medium didn’t cause significant changes of antimicrobial activity (Fig. 3). So, the results of these studies allow us to conclude that Mg ions had more inducible effect on antimicrobial activity of LAB isolates than Ca ions.

### The effects of Ca and Mg ions on antibacterial activity of LAB associations

It was also interesting to study the effect of divalent cations of Ca and Mg, as well as their combination on antimicrobial activity of different LAB associations (mixture of 2 different strains at the ratio 1:1).

**Fig. 4:**
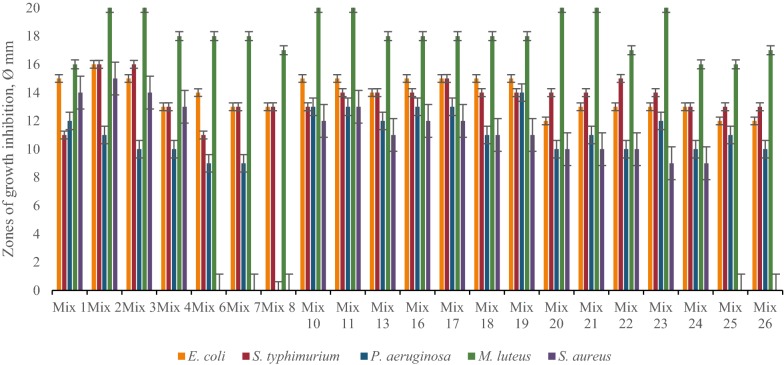
The effect of Ca^2+^ on antibacterial activity of LAB associations (time-spaced cultivation)

**Fig. 5:**
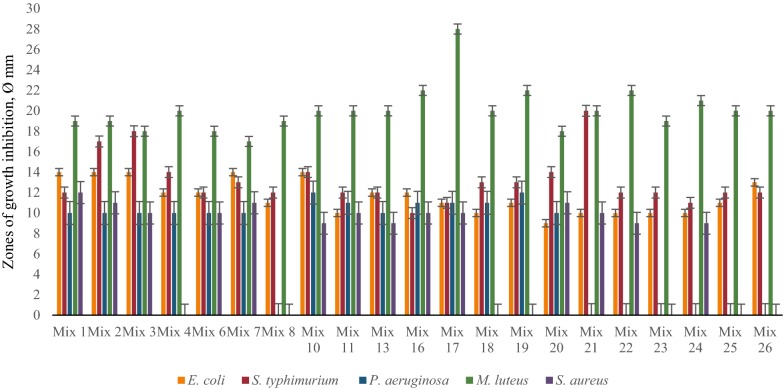
The effect of Mg^2+^ on antibacterial activity of LAB associations (time-spaced cultivation)

**Fig. 6:**
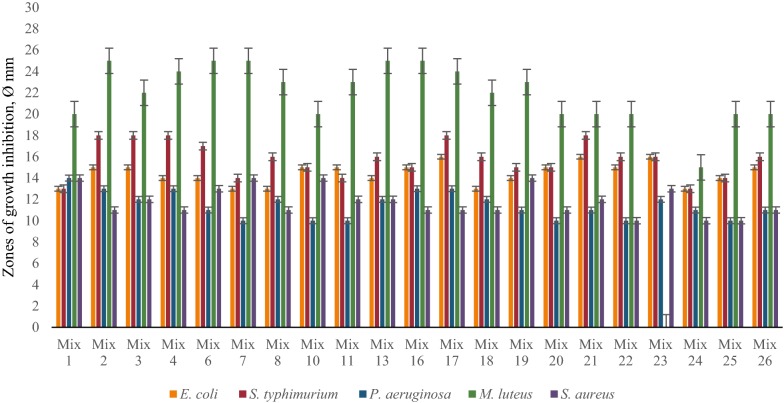
The effect of combined mixture of Ca^2+^ and Mg^2+^ on antibacterial activity of LAB associations (time-spaced cultivation)

**Fig. 7:**
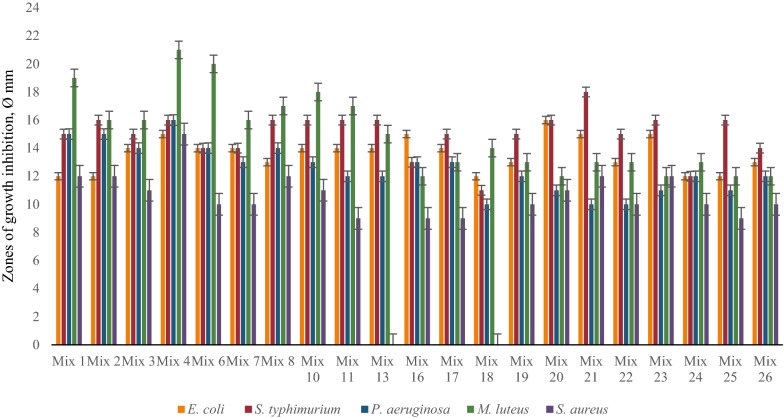
The effect of Ca^2+^ on antibacterial activity of LAB associations (simultaneous cultivation)

**Fig. 8:**
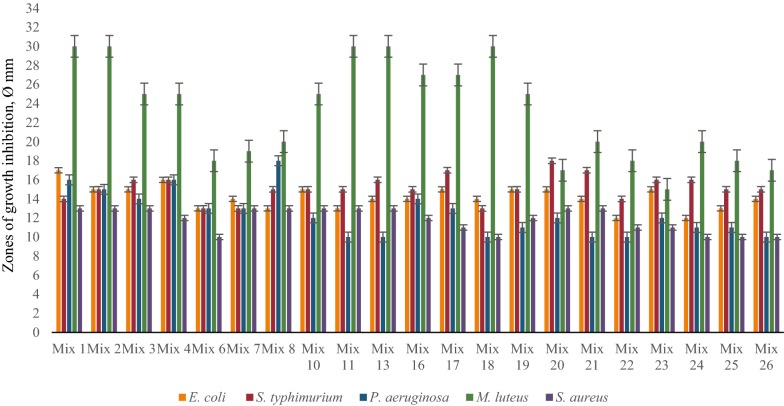
The effect of Mg^2+^ on antibacterial activity of LAB associations (simultaneous cultivation)

**Fig. 9:**
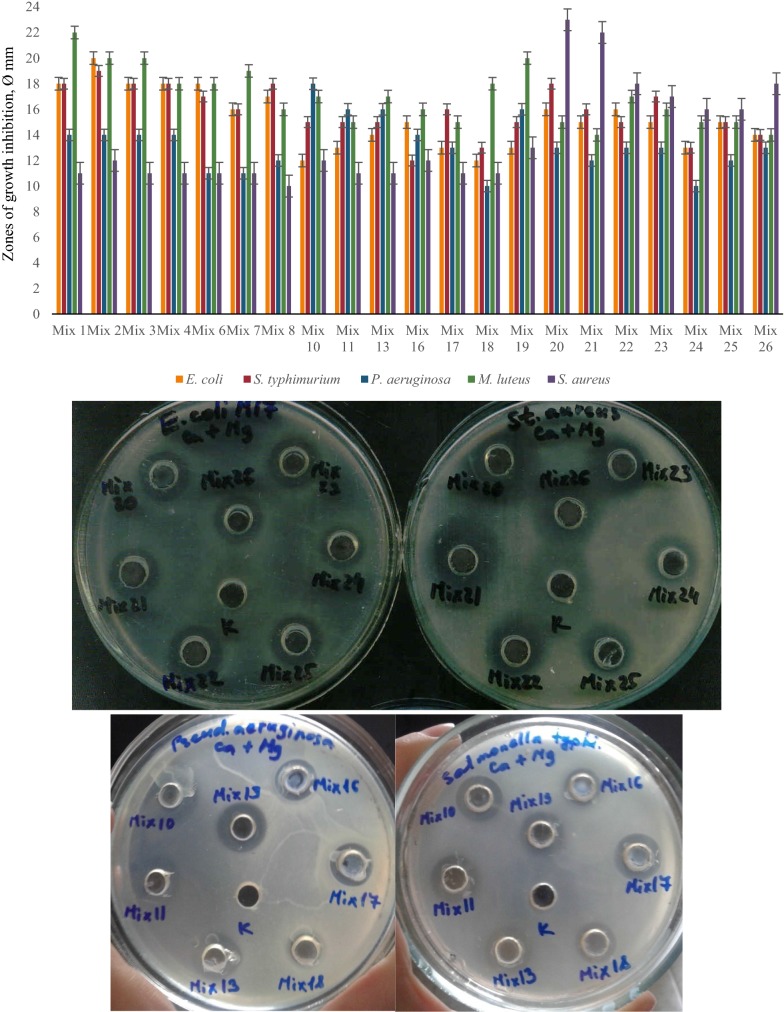
The effect of combined mixture of Ca^2+^ and Mg^2+^ on antibacterial activity of LAB associations (simultaneous cultivation)

The results of these investigations showed significant differences depending on type of cultivation. The best concentrations of Ca^2+^ for antimicrobial activity of LAB associations were around 10 mM and for Mg^2+^ 11 mM (determined specifically for each LAB strain and each association). Most LAB communities showed significant antimicrobial effect against *M. luteus, S. typhimurium* and *E. coli* at different Ca^2+^ concentrations, and some communities inhibited the growth of pathogenic *P. aeruginosa* and *S. aureus* in case of time-spaced cultivation (Fig. 4). Interestingly, the presence of Mg ions in LAB growth medium significantly decreased the antimicrobial activity of LAB mixes against pathogenic test-organisms, vice versa the combined mixture of Ca^2+^ and Mg^2+^ essentially increased the inhibitory effect at the same cultivation conditions (Figs. 5, 6). The inhibitory effect of many LAB associations was significantly increased against all test-organisms at different Mg^2+^ concentrations and especially by the addition of combination of Ca^2+^ and Mg^2+^ but the addition of Ca^2+^ to MRS didn’t cause essential changes of antimicrobial activity of LAB associations in case of simultaneous cultivation (Figs. 7, 8, 9). So, the results of these studies allow to conclude that Ca^2+^ and Mg^2+^ and their combination had more inducible effects on antimicrobial activity in case of simultaneous cultivation of LAB isolates.

In this study the well investigated LAB strains were used. The proteinaceous nature of their antibacterial components had been shown. The bacteriocineous nature of antibacterial components synthesized by LAB associations should be confirmed (Movsesyan et al. 2010; Bazukyan et al. 2018). So we can only speculate that in the combinations they synthesized some antibacterial components with proteinaceous nature, too.

## Discussion

Interactions between metals and microorganisms are diverse, but can be divided into 3 major categories: metals essential for metabolism; metals which are accumulated; metals which undergo biochemical transformation (including leaching). Three individual functions were presented: metal ions act as catalytic centers of enzymes; metal ions, not primarily involved in the catalysis, act as binding groups to bring enzyme and substrate together; metal ions maintain physiological control (antagonism with other metals). More recently, other aspects of the role of metal ions in metabolism have been investigated, e.g. the involvement of metal ions in the reactivation of EDTA inhibited proteolytic enzymes from LAB and the narrow tolerance for specific metals in the synthesis of secondary metabolites (Weinberg 1970, 1978). The ionic environment may interfere with bacterial cell walls, especially in Gram-positive bacteria such as *Lactobacillus* and *Streptococcus* which contain teichoic and teichuronic acids (Ellwood and Tempest 1972). The relative affinities of various cations for Gram-positive bacterial cell walls have been reported by Marquis et al. (Marquis et al. 1976).

Calcium is a very important metal for growth and activity of LAB. As a rule, concentration of calcium in milk is approximately 15 mM (Boyaval 1989). But it enhances the growth of LAB depending on their genera and the composition of their growth medium. Particularly, during studies with *L. casei, L. arabinosus*, *Leuconostoc mesenteroides* and *S. faecalis*, only *L. casei* growth was enhanced by calcium addition. Ca^2+^ stimulated early growth of *L. casei* in an amino acid medium and in media containing limiting amounts of serine (Boyaval 1989). It is interesting to note that in the presence of Ca^2+^ short chains were formed, while in the absence of this ion the cells were in longer chains. The results of our experiments showed that Ca^2+^ induced the antibacterial activity of LAB isolates, as well as LAB associations. Interestingly, Ca^2+^ also stimulated the proteolytic activity of some our investigated LAB strains, as it was shown in previous work (Keryan et al. 2014). The proteolytic activity of MDC9632 and MDC9633 strains was detected previously (Keryan et al. 2017). So, we can consider that there is an interesting correlation between stimulation of antibacterial and proteolytic activity by Ca^2+^. So we can speculate that the antibacterial components were produced after proteolysis, i.e. have the proteinaceous nature. But for confirmation of this hypothesis the further detailed investigations are required. Wright and Klaenhammer (1981) showed that Ca^2+^ supplementation of MRS resulted in a morphological transition of *L. acidophilus* from filamentous to bacilloid rods, which were more resistant to freezing. It was also known that Ca^2+^ plays essential role in the cell wall but it is not clear yet. Mills and Thomas (1978) showed that the liberation of proteinase from cell walls of *S. lactis* and *S. cremoris* stopped when CaCl_2_ was added to the buffer, or when the temperature was raised or when the pH reached 5.5. Thomas et al. (1974) thought that Ca^2+^ linked the cell wall and the enzyme, while Exterkate (1979) showed that Ca^2+^ stabilized proteinase activity.

Magnesium is the major divalent cation in all living cells. In bacterial cells, the intracellular Mg^2+^ content is equivalent to 20–40 mM Mg^2+^ (Silver and Clark 1971). In milk, the Mg^2+^ concentration varies from 4.2 to 6.25 mM, depending on the geographic region (Veisseyre 1975). Interestingly, supplementation of milk with 1-2.1 mM Mg^2+^ permitted both a stimulation of growth of *S. thermophilus* and *S. lactis* and a better survival rate of the lactic streptococci (Amouzou et al. 1985). Interesting results about antibacterial properties of Mg^2+^ were obtained by Duane and coauthors (Robinson et al. 2010). Particularly, they investigated antibacterial properties of magnesium against Gram-positive (*S. aureus*) and Gram-negative (*E. coli* and *P. aeruginosa*) bacteria by addition of Mg^2+^ to their growth medium. Authors indicated that Mg metal has a significant effect on CFUs of both Gram-positive and Gram-negative bacteria. Added to the growth media Mg^2+^ corrosion products would inhibit the growth of *E. coli*, *P. aeruginosa* and *S. aureus* but this is only a hypothesis which needs confirmation. In our experiments the increasing of antibacterial activity after addition of Mg ions was approximately as much as after Ca ions addition.

Ca^2+^ and Mg^2+^ have other amazing properties. Particularly, it was shown that these cations can influence on bacterial biofilm formation (Guvensen et al. 2012; Song and Leff 2006). These ions can also interact with antibiotics and protect the bacterial outer membrane from damage (Sahalan et al. 2013).

There are some interesting data about the effects of other metal ions on bacterial cell growth. Particularly, the authors investigated the growth and oxidation–reduction potential of *Enterococcus hirae* in the presence of Mn^2+^ (Vardanyan and Trchounian 2013). They showed that addition of Mn^2+^ (MnCl_2_) within the range of 0.01 to 1 mM affected *E. hirae* growth by decreasing lag phase duration and increasing specific growth rate. Another work was devoted to study of various heavy metal ions effects on bio-hydrogen production and the FoF1-ATPase activity of *Rhodobacter sphaeroides* (Hakobyan et al. 2012). It was shown that Fe^2+^ plays a very important role for growth, hydrogen production and ATPase activity of *R. sphaeroides*.

There is limited data about the effect of ions on the microbial antibacterial activity. Our data showed that some investigated mixed cultures of LAB revealed strong inhibitory effects against pathogenic test-organisms. These associations were Mixes 1, 2, 4, 8, 10, 19 and 20 (see Table 1). Particularly, in case of separated cultivation Mixes 1, 2, 10 and 19 (see Table 1) showed the strongest antibacterial activity against *P. aeruginosa* and *S. aureus.* In case of simultaneous cultivation, the growth of mentioned test-organisms was inhibited by Mixes 4, 8, 10 and 20 (see Table 1). Interestingly, in case of time-spaced cultivation inhibitory effect of mixed cultures was induced by Ca^2+^ and their mixture with Mg^2+^. Antibacterial effect was mostly stimulated by combined mixture of Ca^2+^ and Mg^2+^ in case of simultaneous cultivation. The biggest diameter of growth inhibition zone of *P. aeruginosa* was 18 mm and 23 mm for *S. aureus* caused by Mixes 10 and 20, respectively.

The investigation of effect of Ca^2+^ and Mg^2+^ on growth and biological properties of LAB becomes more prospective. The fact that Ca^2+^ and Mg^2+^ and their mixture stimulated the inhibitory effect of investigated LAB isolates and their associations can play a role for creation of new effective antimicrobial drugs for prevention of pathogens growth. Anyway, the further more detailed investigations are required.

## Abbreviations

LAB: lactic acid bacteria
FIRL: Biopolymers Interaction Assemble, Function and Interaction of Proteins Laboratory
INRA: National Research Institute of Agronomy
MDC: Microbial Depository Center
MRS: De Man, Rogosa and Sharpe
